# Bacterially produced *Pt*-GFP as ratiometric dual-excitation sensor for *in planta* mapping of leaf apoplastic pH in intact *Avena sativa* and *Vicia faba*

**DOI:** 10.1186/1746-4811-10-31

**Published:** 2014-10-02

**Authors:** Christoph-Martin Geilfus, Karl H Mühling, Hartmut Kaiser, Christoph Plieth

**Affiliations:** Institute of Plant Nutrition and Soil Science, Christian-Albrechts-Universität zu Kiel, Hermann-Rodewald-Str. 2, 24118 Kiel, Germany; Botanisches Institut, Christian-Albrechts-Universität zu Kiel, Am Botanischen Garten 3-9, 24118 Kiel, Germany; Zentrum für Biochemie und Molekularbiologie, Christian-Albrechts-Universität zu Kiel, Am Botanischen Garten 3-9, 24118 Kiel, Germany

**Keywords:** GFP, Genetically encoded biosensor, Plant bioimaging, Apoplast, pH, Salinity, Nitrogen forms, Stress, Signaling, *Ptilosarcus gurneyi*, Three-channel ratio imaging

## Abstract

**Background:**

Ratiometric analysis with H^+^-sensitive fluorescent sensors is a suitable approach for monitoring apoplastic pH dynamics. For the acidic range, the acidotropic dual-excitation dye Oregon Green 488 is an excellent pH sensor. Long lasting (hours) recordings of apoplastic pH in the near neutral range, however, are more problematic because suitable pH indicators that combine a good pH responsiveness at a near neutral pH with a high photostability are lacking. The fluorescent pH reporter protein from *Ptilosarcus gurneyi* (*Pt-*GFP) comprises both properties. But, as a genetically encoded indicator and expressed by the plant itself, it can be used almost exclusively in readily transformed plants. In this study we present a novel approach and use purified recombinant indicators for measuring ion concentrations in the apoplast of crop plants such as *Vicia faba* L. and *Avena sativa* L.

**Results:**

*Pt*-GFP was purified using a bacterial expression system and subsequently loaded through stomata into the leaf apoplast of intact plants. Imaging verified the apoplastic localization of *Pt-*GFP and excluded its presence in the symplast. The pH-dependent emission signal stood out clearly from the background. *Pt*GFP is highly photostable, allowing ratiometric measurements over hours. By using this approach, a chloride-induced alkalinizations of the apoplast was demonstrated for the first in oat.

**Conclusions:**

*Pt-*GFP appears to be an excellent sensor for the quantification of leaf apoplastic pH in the neutral range. The presented approach encourages to also use other genetically encoded biosensors for spatiotemporal mapping of apoplastic ion dynamics.

## Introduction

The pH in the aqueous phase of the leaf apoplast controls multiple metabolic processes and is related to signaling cascades [1, 2]. Changing environmental conditions can alter the leaf apoplastic pH, consequently affecting processes that depend upon the apoplastic H^+^ concentration. Among these are the proton motive force driven transport of metabolites and mineral nutrients across the membrane. Equally important is the effect of a changing pH on the protonation state of peptides or proteins [3–7]. The protonation state of amino acid residues can alter the protein’s structure, leading to pH related conformational changes (misfolding) that impair the affinity to binding sites or its function [8]. For hormones that behave according to the anion trap mechanisms for weak acids (e.g. abscisic acid), the state of protonation is of particular importance for the compartmental distribution [9–11] and their affinity to receptors [12].

Biotic and abiotic environmental factors that influence the leaf apoplastic pH include, among others, the nitrogen nutrition [13, 14], the onset of drought, hydric stress, salinity or anoxia [15–21] and the colonisations and associations by e.g. fungal pathogens or mutualistic mycorrhiza [18, 22, 23]. Developmental and physiological processes like acid-growth, light-sensing, gravitropism or the nitrate assimilation in combination with a production of OH^−^ after cytosolic nitrate reduction are related to apoplastic pH changes [24–30]. In this light, the need for methods that enable an *in planta* quantification of leaf apoplastic pH dynamics with a high spatiotemporal resolution becomes evident. Quantitative ratiometric analyses that combine H^+^-sensitive fluorescent dyes with microscopy based imaging techniques represent a suitable approach for spatiotemporal monitoring of pH dynamics [31–34]. However, since pH-fluorophores are not sensitive over the whole physiological pH range that can exist in the leaf apoplast, the technique of ratio imaging has some limitations. Detection of the leaf apoplastic pH value in its full span that ranges from relative neutral (6.5 to 7.0) to more acidic (below 4.0 to 5.0) [1, 27, 35–37] is not possible, because all available ratiometric pH indicators only cover a limited range of approx. 2–2.5 pH units over which pH sensitivity is most dynamic.

For the acidic pH range, the pH-sensitive dextranated fluorescein derivative Oregon Green 488 is well suited because (i) it has a p*K*a of 4.7 at which its pH sensitivity is most dynamic [12] and (ii) it has a tremendous photostability in combination with a good fluorescent brightness [34]. Apoplastic pH measurements in the more neutral pH range that last over hours, however, are more problematic. Besides the requirements for a pH sensitivity in the near-neutral pH range, the dye must be photostable over hours and large enough to avoid migration from the apoplastic space across the plasmalemma membrane into the cytosol. Fluorescein isothiocyanate-based dyes that are chosen for measurements in this rage (pK_a_ of 5.92; [38]) have a low photostability and are prone to photobleaching [39], excluding them from long term measurements. 2′,7′-Bis-(2-carboxyethyl)-5-(6)-carboxy fluorescein (BCECF), another fluorescein-based dye also appears promising as it has a p*K*a of 7.0 [40], but has the disadvantage that it photobleaches relatively quickly [40]. Schulte *et al*. [1] presented a fluorescent pH reporter protein from the orange seapen *Ptilosarcus gurneyi* (*Pt-*GFP) that, when expressed in *Arabidopsis thaliana,* is used as a genetically encoded pH sensor in the relatively neutral cytosol [41]. Due to its good pH responsiveness at neutral pH (pKa of 7.3), *Pt-*GFP is ideal for pH recordings in the near neutral range that prevails in the leaf apoplast of some plant species.

Unfortunately, these self-expressed biosensors can almost exclusively be inserted into plants that are readily transformable. This impairs the usage of these powerful genetically encoded ion sensors in crop research since almost all agricultural relevant plants are not straightforward to transform.

In this study an approach was elaborated that allowed to use *Pt-*GFP for ratiometric analysis of the pH in the leaf apoplast of crops such as field bean (*Vicia faba* L.) and oat (*Avena sativa* L.) that, otherwise, would need to be transformed very laborious.

It was our strategy to purify *Pt-*GFPs from a bacterial expression system and to test whether this ratiometric dual-excitation pH indicator can (i) be non-invasively loaded directly into the apoplast of intact plants through the stomata and whether (ii) *Pt-*GFPs are suitable for detecting stress-related apoplastic pH changes in the near-neutral pH range.

## Material and methods

### Plant cultivation

*Vicia faba* L., minor cv Fuego (Saaten-Union GmbH, Isernhagen, Germany) was grown under hydroponic culture conditions in a climate chamber (14/10 h day/night; 20/15°C; 60/50% humidity; Vötsch VB 514 MICON, Vötsch Industrietechnik GmbH, Balingen-Frommern, Germany) as described in detail by Geilfus and Mühling [37]. The nutrient solution had the following composition: 0.1 mM KH_2_PO_4_, 1.0 mM K_2_SO_4_, 0.2 mM KCl, 2.0 mM Ca(NO_3_)_2_ or as given in the figure legends, 0.5 mM MgSO_4_, 60 μM Fe-EDTA, 10 μM H_3_BO_4_, 2.0 μM MnSO_4_, 0.5 μM ZnSO_4_, 0.2 μM CuSO_4_, 0.05 μM (NH_4_)_6_Mo_7_O_24_. Hydroponic cultivation of *Avena sativa* L. was conducted in an structurally identical climate chamber with the settings and growth conditions given elsewhere [42, 43]. After 10–20 d of plant cultivation, *in vivo* pH recording was performed as described below.

### Bacterial expression of GFPs

*Pt-*GFP (Acc.No. AY015995) was expressed as described in Schulte *et al*. [1] using the bacterial expression vector pRSETb (Invitrogen GmbH; Karlsruhe, Germany). The 6xHis-tagged fluorescent protein was purified and concentrated through a Ni^2+^/NTA-agarose column (Qiagen, Hilden, Germany) followed by gel filtration through a NAP-25 column (Pharmacia Biotech, Freiburg, Germany). The protein was stored frozen in PBS. Before apoplast loading, the *Pt-*GFP proteins were dialysed over night against Mes-buffer (5 mM MES Roth # 4256; 5 mM K_2_SO_4_; Merck # 5153) with a MWCO of 10 kDa.

### Loading of pH indicators into the intact leaf apoplast

For means of *in planta* recording of leaf apoplastic pH values, 7.5 μg/ml of the fluorescent pH indicator *Pt-*GFP or 25 μM of the pH-sensitive dye Oregon Green 488-dextran (Invitrogen GmbH, Darmstadt, Germany) were loaded into the leaf apoplast of intact plants following the step-by-step instructions that were given elsewhere [34]. Measurements were started 2 hours after loading.

### Confocal laser scanning microscopy

To visualize the *Pt*-GFP distribution within the leaf apoplast, CLSM imaging via a Leica TCS SP5 confocal laser scanning system (Leica Microsystems, Wetzlar, Germany) was carried out. For *Pt*-GFP excitation, the 488 nm beam line of an argon laser was chosen. Emission bandwith was 498–540 nm. Chloroplast autofluorescence was excited at 633 nm by a helium-neon laser (emission bandwith was 650 nm–704 nm). A planapochromatic objective (HC PLAN APO 20.0 × 0.70; Leica Microsystems) was used for image collection.

### Image acquisition for *in vivo*pH-recording

Fluorescence images were collected as a time series with a Leica inverted microscope (DMI6000B; Leica Microsystems, Wetzlar, Germany) connected to a DFC camera (DFC 360FX; Leica Microsystems) via 5-fold magnification (0.15 numerical aperture, dry objective; voxel size = 0.002 mm; HCX PL FLUOTAR L, Leica Microsystems). An HXP lamp (HXP Short Arc Lamp; Osram, Munich, Germany) was used for illumination at excitation wavelengths 387/11, 440/20 and 490/10 nm. The exposure time was 25 ms for all channels. Emission was collected at 510/84 for both *Pt*-GFP channels and 535/25 for both OG channels using band-pass filter in combination with a dichromatic mirror (LP518; dichroit T518DCXR BS; Leica Microsystems). Plants were supplied with aerated nutrient solution.

### Ratiometric analysis

The fluorescence ratios *F*_490_/*F*_387_ (*Pt*-GFP) and *F*_490_/*F*_440_ (Oregon Green 488) were obtained as a measurement of pH on a pixel-by-pixel basis. Image analysis was carried out using LAS AF software (version 2.3.5; Leica Microsystems). In order to take into account a potential variability in the leaf apoplastic pH that might exist across the imaged leaf detail, ratio image was divided in 6 ROIs per ratio image and time point. Background values were subtracted at each channel. For conversion of the fluorescence ratio data gained with the Oregon Green dye into apoplastic pH values, an *in vivo* calibration was conducted. In brief, Oregon Green dye solutions were pH buffered and loaded into the leaf apoplast. The Boltzmann fit was chosen to fit sigmoidal curves to the calibration. Fitting yielded an area of best responsiveness in the range pH 3.9–6.3 for the Oregon Green dye [34]. When the leaves were loaded with pH buffer, all regions of the apoplast showed the same ratio signal at the same buffered pH. Despite this uniformity, the absolute pH values quoted should be viewed as approximations of the apoplastic pH [44], because we cannot exclude the possibility that the buffer reaches equilibrium with the steady-state pH environment within the leaf. Nevertheless, this does not preclude a biological interpretation of leaf apoplastic pH responses to experimental treatments, because it was demonstrated that manipulation of the PM proton pump ATPase (PM-H^+^-ATPase) activity with fusicoccin or vanadate lead to the expected effects on the apoplastic pH as measured by a ratiometric dye [37]. For pseudo-color display, the ratio was color-coded ranging from purple (no signal) over blue (lowest detectable pH signal) to pink (highest detectable pH signal). The *Pt*-GFP ratio signal was calibrated following the same procedure using citric acid/sodium citrate (3.5 ≤ pH ≤ 5.5; 10 mM), MES (5.5 ≤ pH ≤ 6.5; 50 mM), PIPES (6.0 ≤ pH ≤ 7.5; 50 mM), HEPES (7.0 ≤ pH ≤ 8.5; 50 mM) and TRIS-base/MES (8.5 ≤ pH ≤ 10.5; 50 mM).

## Results and discussion

Plants respond to stress through complex signaling networks that regulate and coordinate transcriptional and physiological processes initiating adaptations that help the plant to endure under unfavorable conditions [45] and references therein; [46] and references therein. Choi *et al.* [46] report in accordance to other authors [2, 36, 47, 48] that apoplastic pH is one of the key factors in transmitting information regarding stress to distant unaffected plant organs [12, 18]. After all, alterations in pH have an impact on protein folding, hormone distribution, channel and transporter activity or on membrane integrity and traffic [12, 49].

There is increasing evidence that pH dynamics in the apoplast are involved in stress perception and systemic communication [2, 20, 45, 50]. To better understand the role of transient pH dynamics, the need for indicators that allow the detection of apoplastic pH in its full span ranging from relative neutral to more acidic becomes evident. While the more acid range can easily be covered by the dextranated dye Oregon Green 488, there is lack in photostable dyes that allow ratiometric dual-excitation measurements in the relatively neutral range.

The fluorescent pH reporter protein from the orange seapen *Ptilosarcus gurneyi* (*Pt-*GFP) may provide a solution since it has a very broad pH-responsiveness that also covers relatively neutral pH values and an excellent dynamic ratio range [1]. However, it has the drawback that this genetically encoded pH sensors can only be used in plants that can be genetically transformed. For this reason, the abundance of self expressed biosensors which is currently available cannot readily be used for some agricultural crop plants without considerable expenditure. In order to make these valuable indicators available, we tested an approach for non-invasively loading bacterially produced *Pt*-GFP into the leaf apoplast of intact plants. In order to test the suitability of this strategy, leaf apoplastic pH dynamics were induced by salt stress at the roots of field bean (*Vicia faba* L.) and oat (*Avena sativa* L.).

### Localisation of leaf apoplastic loaded *Pt*-GFP

**Figure 1:**
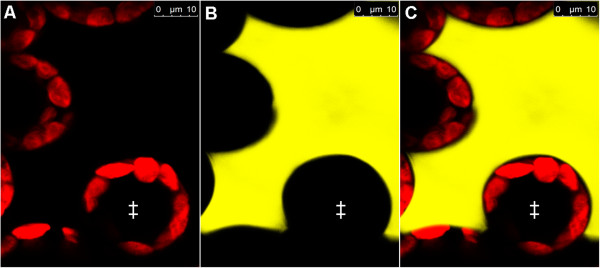
**Apoplastic distribution of the** ***Pt*** **-GFP in a** ***Vicia faba*** **leaf infiltrated 2–4 minutes prior image acquisition.** *Pt* GFP is exclusively located in the apoplast. Confocal image in **(A)** shows adaxial view on palisade cell chloroplasts (exited at 633 nm; pseudo-red). Image in **(B)** shows same detail with *Pt*-GFP (excited at 488 nm; pseudo-yellow). **(C)** Overlay of **(A)** and **(B)** demonstrates that the *Pt* GFP is only located in the apoplast. No *Pt* GFP signal is emitted from between the chloroplasts, indicating that the *Pt*-GFP did not enter the cytosol. Moreover, the inside of the palisade cells remained black, proving that *Pt*-GFP did not enter the vacuole or other symplastic organelles, as otherwise signals would be detectable from the cells. Symplastic *Pt* GFP location was negated in several leaves derived from different plants. ‡, palisade cells.

**Figure 2:**
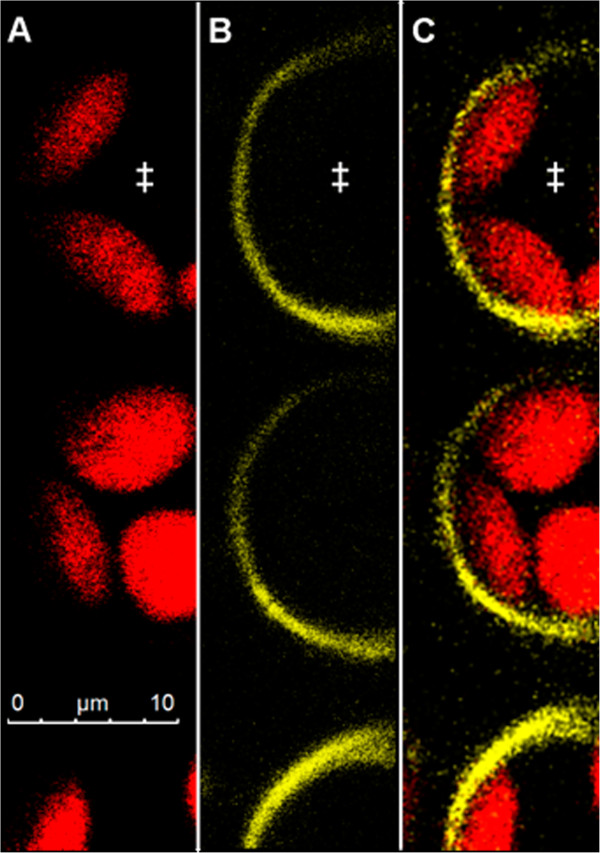
**Apoplastic distribution of the** ***Pt*** **-GFP in a** ***Vicia faba*** **leaf infiltrated 2.5 h prior image acquisition.** *Pt* GFP is exclusively located in the apoplast. Confocal image in **(A)** shows adaxial view on palisade cell chloroplasts (exited at 633 nm; pseudo-red). Image in **(B)** shows same detail with *Pt*-GFP (excited at 488 nm; pseudo-yellow). **(C)** Overlay of **(A)** and **(B)** verifies the apoplastic distribution of the *Pt*-GFP that is attached outside of the palisade cells and, by this means, outlines the cell boundaries at 2.5 hours after loading. No *Pt*-GFP signal is emitted from between the chloroplasts, proofing that no *Pt*-GFP had entered the cytosol. Symplastic *Pt* GFP location was negated in several leaves derived from different plants. ‡, palisade cells.

Studies on leaf apoplastic ion concentrations require an ion indicator that is reliably localized in the apoplast and not unintentionally in cellular compartments, the cytosol or the vacuole. Otherwise, signals from the e.g. neutral cytosol or the very acidic vacuole would affect the apoplastic pH estimations. Additionally, it must be ensured that the bacterially produced *Pt-*GFP proteins can be inserted into the leaf apoplast by liquid mass flow through stomatal pores, following the protocol to which was referred in the material and methods-section. To visualize the indicator’s localization after apoplast loading, CLSM imaging was carried out. Confocal imaging (Figure 1) revealed that the *Pt-*GFP could easily be loaded into the apoplast following the protocol described by Geilfus and Mühling [34]. Moreover, images proved that *Pt*-GFP had not unintentionally entered the symplast at 2–4 minutes after loading, as otherwise signals would be detectable from the cells. It is very likely that the size of the *Pt*-GFP that is approximately 105 kDa in its native form [1] ensured that it did not access the symplast by crossing the plasma lemma from the apoplast. Images in Figure 2 demonstrated that in longer periods of time in which experiments are conducted, e.g. 2.5 hours after loading, the *Pt-*GFP still maintained to be exclusively apoplastically located. Moreover, it can be seen that the apoplast is not flooded (and thus not anoxic) during measurements and that *Pt-*GFP behaves like Oregon green because it is attached outside of the palisade cells as it was previously observed for the fluorescent dye ([37], Figure number eight therein).

### Background and photostability

**Figure 3:**
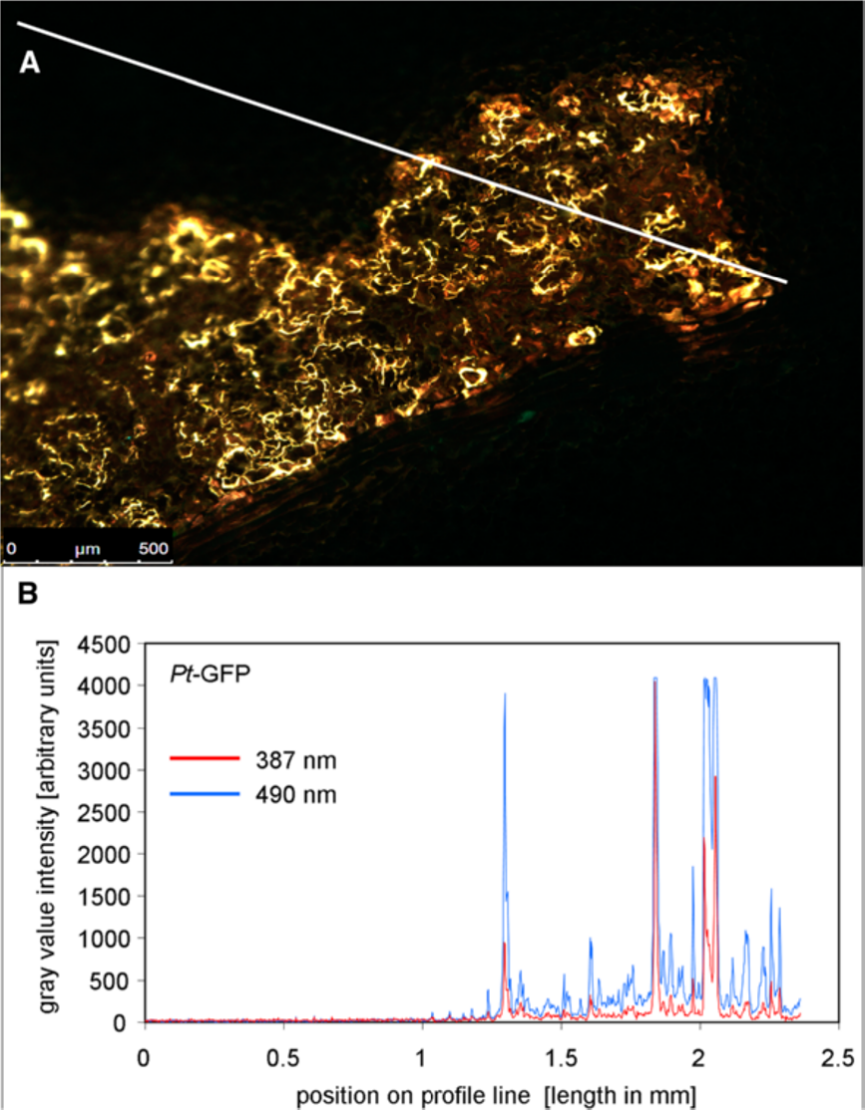
***Pt*** **-GFP emission signals are markedly higher than unspecific background signals. (A)** Overlay of ex 490 nm (pseudo-green) and ex 387 nm (pseudo-blue) fluorescence images shows adaxial leaf apoplast that was partially loaded with the *Pt*-GFP. Under illumination, dye-loaded areas appear yellowish because pseudo-green and pseudo-blue are mixed within the overlay. Areas that were not loaded with the pH reporter protein appear black when illuminated and serve to compare the amount of unspecific signals (background) to signals that are specifically emitted from the proteins. For this comparison, the intensity of grey values was chosen as a measure for signal intensity. A profile of the intensity values was taken from the white line in **(A)** and is presented in **(B)**. Profiles are displayed for both fluorescence excitation channels (*F*_490_ and *F*_387_). Only negligible signals were emitted from the area without dye and signals were much higher in the dye-loaded areas. Twelve separate images captured from different plants proved the low background intensity.

*In planta* measurements of apoplastic ion dynamics using microscopy-based ratio analysis require a signal-to-background ratio that is large enough to coherently reflect changes in the analyte concentration in the natural environment of the specimen. Background is all the light in the optical system that is not specifically emitted from the pH sensors and, if not considered, might introduce errors in quantitation. Background signals sum up from autofluorescence coming from the measuring devices (i.e., lens elements), the specimen (i.e., chloroplasts or cell wall compounds such as oxidized phenols), the shot background associated with sampling of the signal [32, 51], and the avoidable background arising from residual light in the laboratory (i.e., computer LEDs, monitor screens). In order to evaluate whether the signal-to-background ratio of the *Pt*-GFP is large enough for ratio analysis, the specimen without the dye was illuminated (background signal intensity) and was compared with the specimen plus dye (signal intensity). In result, only negligible background was detectable (Figure 3; less than 1‰ of the weakest fluorescence signals). The emission signal stood out clearly from the background. This test revealed that the emitted analyte signal was strong enough to allow ratiometric analysis.

**Figure 4:**
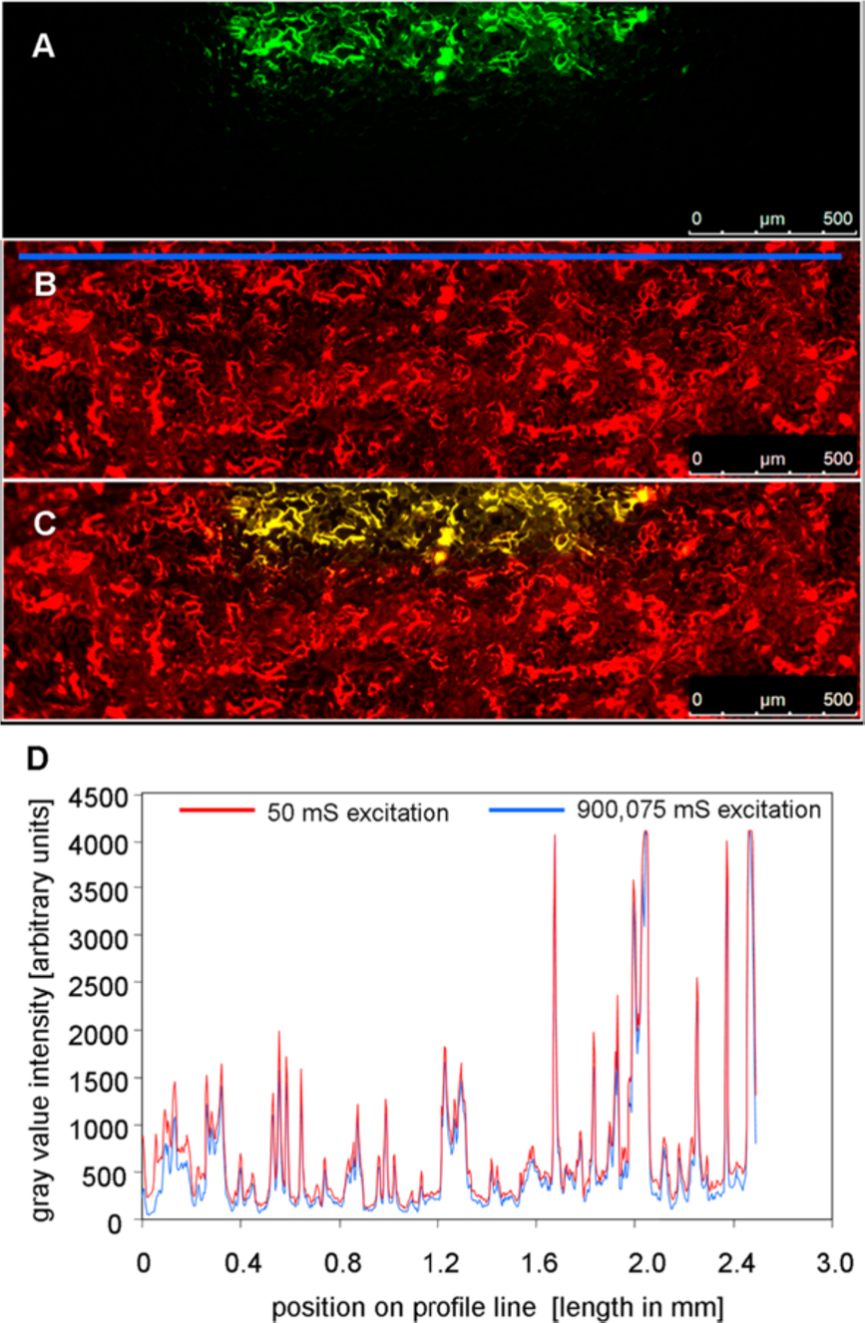
***Pt*** **-GFP is photostable. (A)** The leaf apoplast of *Vicia faba* was loaded with *Pt*-GFP. To test whether *Pt*-GFP is prone to bleaching, a selected area (pseudo-green) was designated to be continuously excited by 490 nm illumination over a period of 15 min (=900,000 ms). The outer edges of the specimen were protected against continuous illumination by foreclosing the field diaphragm (non-bleached are appears black). Prior bleaching was started, initial signal intensity of the specimen was documented (image not shown). **(B)** After 900,000 ms continuous excitation, the field diaphragm was opened for collecting an image at ex 490 nm (exposure time was 25 mS). The image is presented in pseudo-red and contains the part of the specimen that was continuously illuminated (in total 3*25 ms illumination from three image acquisitions plus 900,000 ms from bleaching treatment) plus the area of the specimen that was not bleached (exposed in total to 2*25 ms illumination from two acquisitions). **(C)** Merged overlay of **(A)** and **(B)**. The yellow area (mixing pseudo-red and pseudo-green yields orange) represents the part that was continuously exposed to light treatment (in total 900,075 ms) and, thus, contains the possibly bleached proteins. Pseudo-red area represents the non-bleached part of the leaf with only 50 ms illumination in total (due to image acquisition cycles). Image **(B)** was used to create a profile of the emission intensity values from the area tagged by the blue line as a measure for the photostability. This line covers the bleached and non-bleached areas. The intensity values are presented in **(D)**. A comparison of the intensity values derived from the bleached and non-bleached areas revealed that no significant bleaching occurred after 15 min of continuous illumination. Eight separate bleaching experiments proved photostability of *Pt*-GFP.

Besides a large signal-to-background ratio, the *Pt*-GFP needs to have a high photostability allowing to record pH dynamics over a period of several hours without (photo-) bleaching. In order to test whether *Pt*-GFP was prone to bleaching, the leaf apoplast was loaded with *Pt*-GFP proteins and continuously excited by 490 nm illumination over a period of 15 min. Subsequently, signal emission intensity was compared between bleached and non-bleached areas. This comparison revealed that no dye-bleaching had occurred to a relevant extent after 15 min of continuous illumination (Figure 4), which is satisfactory because in the present work leaves were illuminated about approximately 4000 ms in maximum. *Pt*-GFP was extremely photostable under continuous *F*_490_-light exposure (same was true for the *F*_387_-light channels; data not shown). This means that *Pt*-GFP is suitable for hours of pH recording in the apoplast of intact plants.

### *Pt*-GFP as leaf apoplastic pH indicator

**Figure 5:**
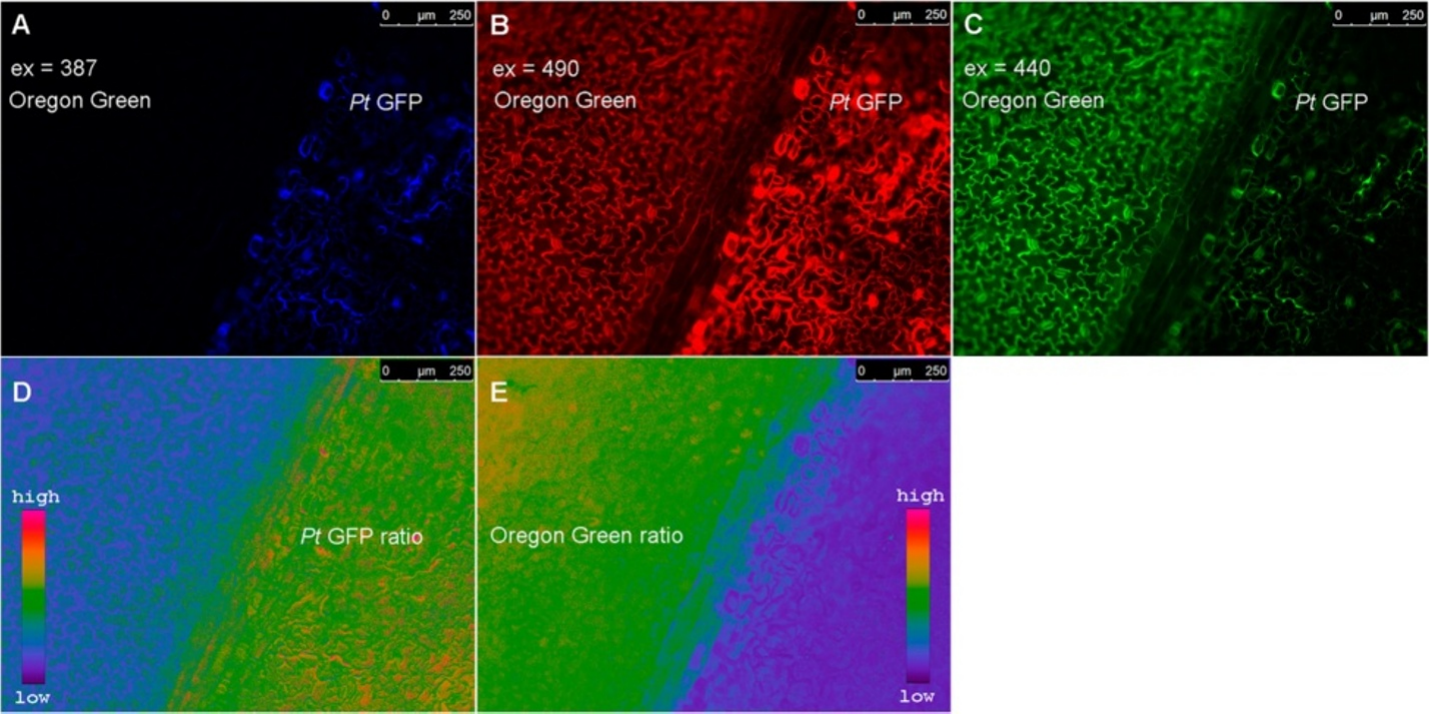
**Principle of ratiometric analysis using two pH indicators within a single image frame.** Fluorescence images shows adaxial view of *Vicia faba* leaf as excited at **(A)** *F*_387_, **(B)** *F*_490_ and **(C)** *F*_440_. Leaf apoplast as loaded with the pH-indicator protein *Pt*-GFP (right of leaf vein) and the pH-indicator dye Oregon Green-dextran 488 (left of leaf vein). Images were captured approximately 3 hours after loading. The fluorescence ratios **(D)** *F*_490_/*F*_387_ (*Pt*-GFP) and **(E)** *F*_490_/*F*_440_ (Oregon Green 488-dextran) were obtained as a measurement of pH. Emission was collected at 510/84 for both Pt-GFP channels and 535/25 for both OG channels. For this reason, the *F*_490_ channel was captured two times, once with emission 510/84, then with emission 535/25 (only the 535/25 emission image is shown in this figure). The ratios were coded by hue on a spectral colour scale ranging from purple (no signal) to blue (lowest signal) to pink (highest signal). Following this new loading strategy, leaf regions loaded with different indicators could be monitored and ratios were calculated according to the optimal wavelength of the respective indicator.

**Figure 6:**
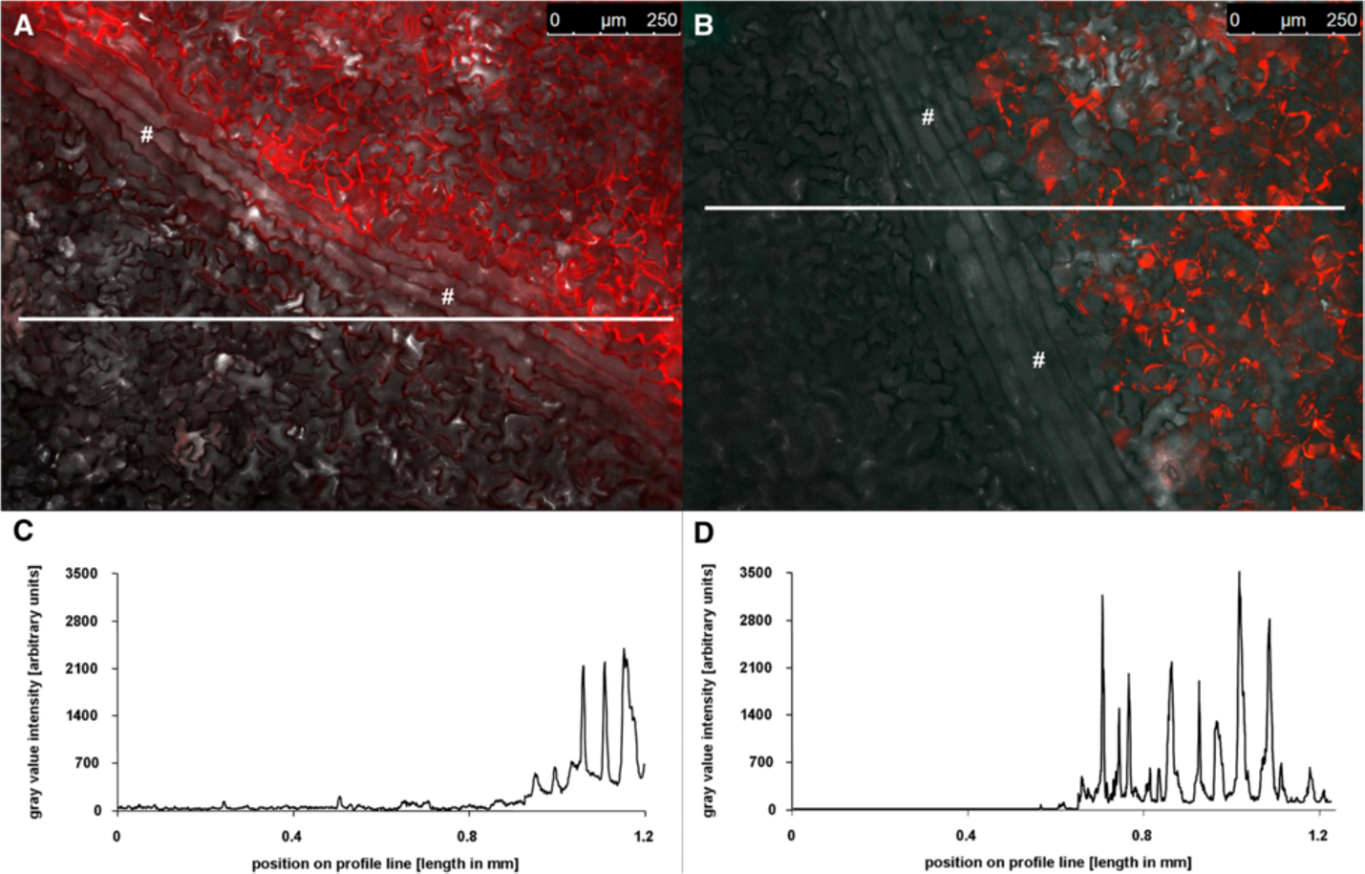
**Leaf vein as a structural barrier that separates apoplastically located dyes.** Adaxial leaf apoplast partially loaded with **(A)** Oregon Green 488-dextran or with **(B)** *Pt*-GFP. Overlay of pseudo-red fluorescence image at F_490_ and corresponding bright field image captured approximately 3 hours after loading. Dye-loaded areas in **(A)** and **(B)** appear red. Areas that were not loaded with the fluorescent pH reporter appear grey when illuminated and serve as a suitable area to compare the amount of unspecific signals (background) to specific signals being emitted from the pH reporters. For this, the intensity of grey values was chosen as a measure for emitted signal intensity. A profile of the intensity values was taken from the white line in **(A)** and is presented in **(C)**. The same was done for the *Pt*-GFP: The intensity values were taken from the white line in **(B)** and are presented in **(D)**. Only negligible signals were emitted from the areas without pH indicator that were separated by the leaf vein from the loaded apoplast. Signals were markedly higher in the dye-loaded areas. #, leaf vein. Results were confirmed by 10 replicates captured from different plants.

**Figure 7:**
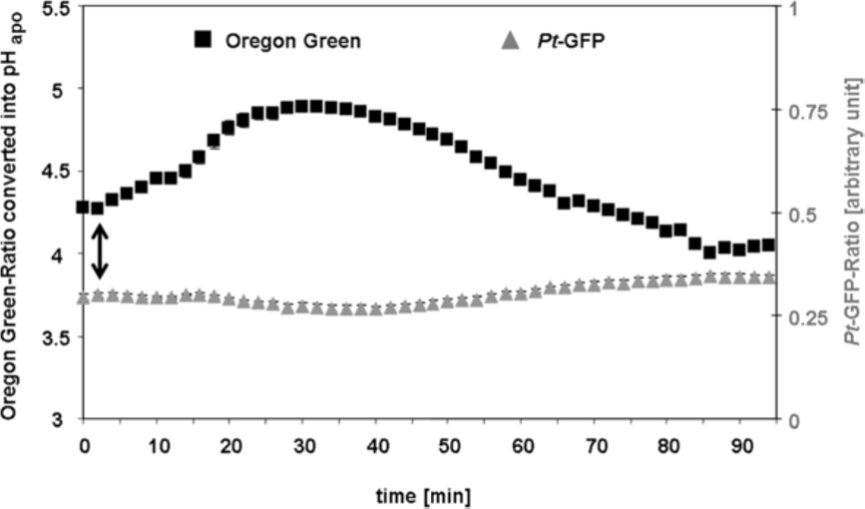
**Unsuitability of** ***Pt*** **-GFP in the acid leaf apoplast of** ***Vicia faba*** **.** Comparison between the responsiveness of the pH indicator protein *Pt*-GFP (grey) and the pH indicator dye Oregon Green (black) to apoplastic pH changes as induced by the addition of 50 mM Cl^−^ via L-cysteinium chloride to the roots of *Vicia faba*. Time point of chloride addition is indicated by the arrow. pH, as quantified at the adaxial face of *Vicia faba* leaves is plotted over time. Fluorescence ratio data obtained by *Pt*-GFP were below the linear range of the *in vivo* pH calibration and, therefore, could not be converted into pH data. Leaf apoplastic pH quantification was averaged (n = 6 ROIs per ratio image and time point; mean ± SE of ROIs). Representative kinetics of eight equivalent recordings of plants gained from 8 independent experiments.

**Figure 8:**
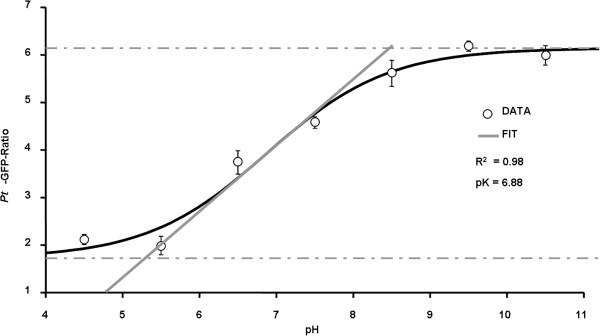
***In vivo*** **calibration of** ***Pt*** **-GFP fluorescence ratio (F**_**490**_ **/F**_**387**_ **).** The Boltzmann fit was chosen for fitting sigmoidal curves to calibration ratio data. Fitting resulted in an optimal dynamic range for pH measurements between 5.3 and 8.4. *In vivo* calibration was conducted on six different plants, each biological replicate was technically replicated. Data are mean of n = 6 ± SE.

The suitability and responsiveness of apoplastically loaded *Pt*-GFP to pH changes was evaluated by a comparison to the established fluorescent pH indicator Oregon Green 488-dextran. In order to enable a proper comparability, measurements were conducted simultaneously side by side within the apoplast of the same field bean leaf. For this purpose, the *Pt*-GFP was loaded adjacent to a region loaded with Oregon Green (Figure 5A-C). The leaf veins clearly separated the GFP proteins from the dye and thus prevented the mixture of both H^+^-indicators (Figure 6). Thereby, leaf regions loaded with different indicators could be monitored within a single image frame and ratios were calculated according to the optimal wavelength of the respective indicator (Figure 5D-E). Following this loading strategy, the dynamics of two different ions/analytes could be visualized simultaneously in the identical leaf and *in planta* by “*three-channel ratio imaging*”. For the sake of comparison, pH changes in the leaf apoplast were specifically induced in a controlled manner by adding chloride into the nutrient solution harbouring the roots. This was done because chloride is carried from the roots to the shoot where it probably primes a systemic transient alkalinization in the leaf apoplast [21, 34]. As visualized by the Oregon Green dye, the addition of 50 mM Cl^−^ via L-cysteinium chloride into the nutrient solution resulted in the expected transient leaf apoplastic alkalinization (Figure 7, black kinetic) that might reflect the action of a Cl^−^/*n*H^+^ symporter. A chloride symport across the PM [52–54] possibly results in a decrease of the leaf apoplastic [H^+^] caused by the co-transfer of protons together with chloride anions from the apoplast into the cytosol. However, the *Pt-*GFP ratios did not reflect this alkalinization from pH 4.3 to 5.0 that was indicated by the peak in the Oregon Green ratios (Figure 7, grey kinetic). It seems that the apoplastic pH in field bean leaves was below the range of best responsiveness for the engineered *Pt-*GFP which ranges from approx. 4.0 to 8.0 as measured by Schulte *et al*. [1] *in vitro* with a fluorescence spectrometer and organic buffers adjusted to the desired pH. This raises the question as to whether *Pt*-GFP is still functional and sensitive to high proton concentrations when used *in vivo*. In order to conduct an *in planta* calibration with the aim to test whether the *Pt*-GFP reacts *in vivo* on pH increments from pH 4.5 to 5.0, we buffered the *V. faba* apoplast to pH values ranging from 4.5 to 10.5 in increments of 0.5 pH units. It turned out that a pH below 5 can not be measured *in vivo* with the *Pt*-GFP (Figure 8), finally explaining the discrepancies in the comparative measurement presented in Figure 7. Based on the *in vivo* calibration, only pH changes ranging from values > 5 to 8 can be monitored.

### Nitrate nutrition alkalizes the leaf apoplast of *Vicia faba*L

**Figure 9:**
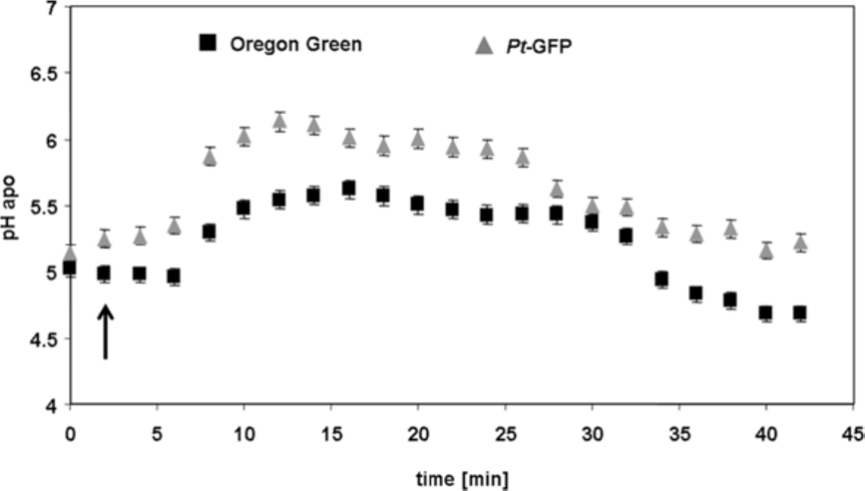
**Nitrate nutrition alkalized the leaf apoplast into** ***Pt*** **-GFP’s range of responsiveness.** Comparison between the responsiveness of *Pt*-GFP (grey) and Oregon Green (black) to apoplastic pH changes as induced by the addition of 25 mM Cl^−^ via L-cysteinium chloride to the roots of *Vicia faba*. Time point of chloride addition is indicated by the arrow. Plants were cultivated with 15 mM nitrate in the nutrient solution given as Ca(NO_3_)_2_. pH as quantified at the adaxial face of *Vicia faba* leaves is plotted over time. Leaf apoplastic pH was averaged (n = 6 ROIs per ratio image and time point; mean ± SE of ROIs). Representative kinetics of six equivalent recordings of plants gained from independent experiments (n = 6 biological replicates).

In a next experiment, the leaf apoplastic pH was alkalized by increasing the nitrate concentration in the nutrient solution from 4 mM up to 15 mM nitrogen (Figure 9). This nitrogen form-related nutrition increased the permanent apoplastic pH from approx. 4.5 up to approx. 5.0 (compare initial pH in Figures 7 and 9). In this way, the leaf apoplastic pH was lifted to the range of best responsiveness for *Pt*-GFP. The subsequent addition of 20 mM Cl^−^ via L-cysteinium chloride into the nutrient solution resulted in the expected transient apoplastic alkalinization as reflected by the Oregon Green fluorescent dye (Figure 9, black kinetik). This transient alkalinization was also indicated by the *Pt*-GFP (Figure 9, grey kinetik). However, the absolute pH values measured with Oregon Green and *Pt*-GFP differed at the maximum peak height. It is possible that e.g. the cell wall did somehow modify the responsiveness of one of the dye system to pH. Another explanation could be that the indicators are localized in different regions of the apoplast were slightly different pH values prevail [20, 21]. Nevertheless, this does not detract from the interpretation of the effects of Cl^−^-treatment on leaf apoplastic pH because both indicators uniformly recorded the chloride-induced transient leaf apoplastic alkalinization.

The experiments presented in Figures 7 and 9 demonstrated that under conditions of 4 mM nitrate fertilization the leaf apoplastic pH was too acidic, so that the *Pt*-GFP did not act in a range of good responsiveness, possibly due to a fluorescence quench at all wavelengths that caused irreversible conformational changes due to too low pH [1]. Increasing nitrate concentration in the nutrient solution of the beans and the associated alkalinization of the extra cellular space that is partially known to be caused by a nitrate cotransport with H^+^ across the PM [13, 14] increased the leaf apoplastic pH to a range that can be monitored with the *Pt*-GFP.

### *Pt*-GFP as apoplastic pH indicator in *Avena sativa*L.

**Figure 10:**
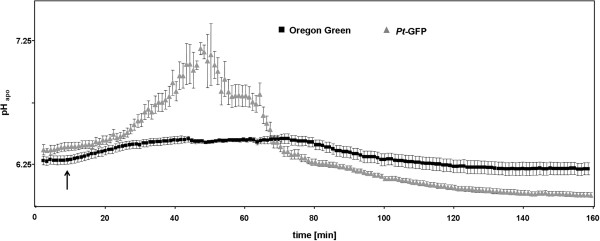
**Near neutral leaf apoplast in** ***Avena sativa*** **requires** ***Pt*** **-GFP for detecting alkalizing effects.** Leaf apoplastic pH response of *Avena sativa* as provoked by the addition of 25 mM Cl^−^ via L-cysteinium chloride into the nutrient solution. Time point of chloride addition as indicated by the arrow. pH, as quantified at the adaxial leaf face is plotted over time. *Pt*-GFP, grey curve; Oregon Green, black curve. Plants were cultivated with 15 mM nitrate in the nutrient solution given as Ca(NO_3_)_2_. Leaf apoplastic pH was averaged (n = 6 ROIs per ratio image and time point; mean ± SE of ROIs). Representative kinetics of eight equivalent recordings of plants gained from independent experiments (n = biological replicates).

Once oat (*Avena sativa* L.) with its less acidic apoplast [35, 55] was chosen for analyzing the formation of the NaCl-induced leaf apoplastic pH peaks, the acidotropic [56] Oregon Green 488 dye seemed to be the wrong choice: Regardless of the fact that the leaf apoplastic pH response was challenged by the addition of 25 mM Cl^−^ given via L-cysteinium chloride into the nutrient solution, the expected transient alkalinization was not reflected by the Oregon Green ratio (Figure 10). It appears that the Oregon Green ratios has reached a ‘plateau phase’ that cannot be exceeded. In contrast, the *Pt*-GFP ratio clearly reflected the transient alkalinization by peaking from an initial pH of approx. 6.25 up to a neutral pH of almost 7.25 (Figure 10). This clearly showed that knowledge about the prevailing pH is mandatorily necessary for choosing the suitable pH indicator. If, for example, Oregon Green would be selected as pH indicator under near neutral conditions, an alkalizing response of the biological system to the stimulus could not be detected and would be misinterpreted as being absent. The experiment has shown that the chloride induced transient alkalinizations which are thought to play a role in the root-to-shoot communication of salt stress [19] are also present in the leaf apoplast of oat. This new finding delivers further indication that the salt-stress induced pH response may occur universally in different plant species.

### Bypassing the cellular secretion pathway

The expression of genetically encoded pH sensors in plants that are targeted to the apoplast is accompanied by the problem that the proteins have to pass the cellular secretion pathway. From here they may also emit fluorescence. These signals from the cytosol, the ER or from Golgi vesicles average with signals from the acid apoplast and may thus lead to incorrect apoplastic pH values [1]. Since there is a pronounced proton gradient between cytosol and apoplast, this problem cannot be neglected. The presented approach of infiltrating bacterially produced and purified pH-sensitive *Pt*-GFP proteins through the stomatal pores into the leaf apoplast bypasses this problem. Moreover, the new approach describes how to use fluorescent reporter proteins for the measurement of leaf apoplastic ion relations in plants that are not readily transformable such as some of the agricultural relevant crop plants. In the light of the increasing availability of self-expressed biosensors [12, 36, 46, 57–64], the presented approach should be seen as example and could also be applied to other genetically encoded sensor proteins or synthetic dyes for spatiotemporal mapping of ion relationships in the intact apoplast.

### Concluding remarks

In summary, confocal laser scanning imaging showed that the tested loading technique is suitable for inserting bacterially produced *Pt-*GFPs into the apoplast of intact plants and negates the unintended presence of the *Pt-*GFP proteins in the symplast. The emission signal stood out clearly from the background and the *Pt*-GFP was characterized by a high photostability allowing ratiometric dual-excitation measurements over hours. *Pt-*GFP appeared to be a very good choice for the *in planta* quantification of leaf apoplastic pH-dynamics in plants that exhibit a relative neutral apoplastic pH. By using this approach, it was found that chloride-induced alkalinizations are not only formed in field beans [20] but also in oat. A strategy is presented that explains how to use bacterially expressed biosensors for the ratiometric *in planta* quantification of apoplastic ion kinetics in real time.

## Abbreviations

CLSM: Confocal laser scanning microscopy
GFP: Green fluorescent protein
Pt: *Ptilosarcus gurneyi*
PM: Plasma membrane
ROI: Region of interest
OG: Oregon Green.
